# Insights into the Dead Sea Transform Activity through the study of fracture-induced electromagnetic radiation (FEMR) signals before the Syrian-Turkey earthquake (M_w_-6.3) on 20.2.2023

**DOI:** 10.1038/s41598-024-54935-8

**Published:** 2024-02-25

**Authors:** Shreeja Das, Vladimir Frid, Avinoam Rabinovitch, Dov Bahat, Uri Kushnir

**Affiliations:** 1 The Department of Civil Engineering, Sami Shamoon College of Engineering, Ashdod Campus, 77662 Ashdod, Israel; 2 Physics Department, Ben-Gurion University of the Negev, P.O.B. 653, 8410501 Beer-Sheva, Israel; 3 Department of Earth and Environmental Sciences, Ben-Gurion University of the Negev, P.O.B. 653, 8410501 Beer-Sheva, Israel

**Keywords:** Natural hazards, Solid Earth sciences

## Abstract

Observations of fracture-induced electromagnetic radiation (FEMR) were conducted along the Dead Sea Transform (DST) from Sodom to Jericho, coinciding with a magnitude (M_w_) 6.3 aftershock earthquake (EQ) in the Turkey-Syrian region on February 20, 2023. The FEMR parameters (“hits,” Benioff strain release, frequency, rise-time, energy) and associated crack dimensions were analyzed, focusing on trends leading up to the EQ. This study investigated the Benioff Strain plot and other parameters in three consecutive earthquake nucleation stages leading to the catastrophe. The first stage showed increased FEMR hits and frequency, decreased rise time (T′), and crack dimensions. In the second stage, FEMR hits and crack width decreased while other parameters continued to rise, accumulating the second-highest energy, likely due to high-stress drop. The third stage exhibited steadily increasing FEMR hits and energy and a notable increase in crack dimensions, suggesting an imminent macro failure event. The cyclic trend in FEMR hits indicates alternating periods of high activity and silence, potentially linked to stress changes during crack propagation. Taken shortly before the earthquake, these measurements offer valuable insights into how FEMR parameters vary before seismic events, bridging the gap between lab-scale studies of rock collapses under stress and large-scale failure phenomena.

## Introduction

### Seismicity of the Dead Sea Transform

The Dead Sea Transform (DST) fault system, also termed the Dead Sea Rift, is a series of strike-slip faults that spread from the Red Sea to southeast Turkey (East Anatolian Fault)^1–4^. This fault system accommodates the differential motion and forms a transform boundary between the African and Arabian plates. The African Plate (to the West) and the Arabian Plate (to the East) are moving in a general NNE direction, resulting in a left lateral strike-slip motion in this plate boundary segment owing to the Arabian Plate moving slightly faster. The northern end of the DST eventuates a complex tectonic region of southeast Turkey, where three tectonic plates, i.e., African, Arabian, and East Anatolian Plates (EAP), converge. The Anatolian tectonic plate is constantly under pressure because it is continuously pressed upwards by the Arabian Plate, which squeezes the Anatolian Plate westward for Eurasia with a speed of around 25 mm/year, where it faces friction from the African Plate, which is also moving upwards^1,2,4,5^.

**Figure 1:**
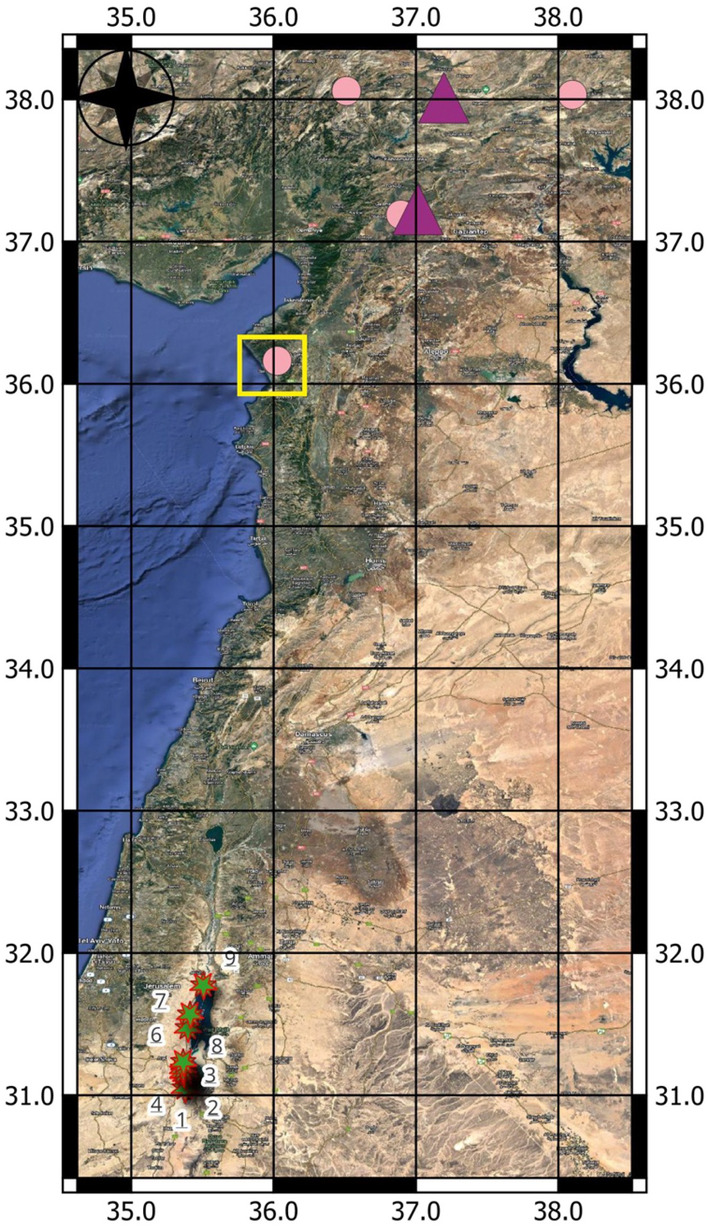
The map of the region of Syrian-African transform with superimposed locations of significant earthquake epicenters related to the Gaziantep Earthquake of 6th Feb 2023 and its subsequent aftershocks^8,9^ (the purple triangles demark the M_w_ > 7, rose circles—M_w_ > 6). This is obtained from the USGS website^7–9^. The highlighted epicenter, demarcated by the yellow square, was the aftershock event at Hatay region, coinciding with our field survey along the Dead Sea Basin^9^. The green stars and numbers (1–9) indicate the location of FEMR monitoring points near the Dead Sea which was obtained during our FEMR survey. The base map on which the earthquake epicenters and the location of the monitoring points have been superimposed is obtained from Google Earth software (https://www.google.com/earth/).

A series of devastating earthquakes in the Turkey-Syrian region occur due to the aforementioned complex tectonism in the area. The most recent and disastrous earthquake of magnitude (M_w_) 7.8 struck southern and central Turkey and northern & western Syria on the 6th of Feb, 2023^6–8^. Its epicenter was 37 km west-northwest of Gaziantep, Turkey, which sits at the collision of two tectonic plates (the East Anatolian Plate and the Arabian Plate). Shortly after the occurrence of this event, an equally disastrous second earthquake of magnitude (M_w_) 7.7 followed, whose epicenter was located around 95 km north-northeast from the first. Following these major events, for the crust to readjust to the changes in stress, approximately 30,000 aftershocks were recorded for the next few months. In fact, on 20th Feb 2023, one of the aftershocks of magnitude (M_w_) 6.3 hit the area of Uzunbag near the Hatay Province, resulting from an oblique normal faulting^9^ (Fig. 1). The catastrophic effects of this earthquake and its aftershock events have caused economic losses for the nations and resulted in fatalities of around 14 million people. This has been one of the most devastating earthquakes in Turkey since the 1939 Erzincan earthquake of M_w_ 7.8^10–14^.

### Geology of the Dead Sea Transform

The Dead Sea Transform (DST) spans 1000 km from the northern Red Sea to the Taurus mountains, originating 18 million years ago^15^. The DST exhibits transtensive and transpressive features involving left lateral slip due to the Arabian Plate’s activity with the African Plate. Its complex motion includes extensional and compressional structures influenced by Proterozoic orogeny and subsequent tectonic events^2,5,15^. The DST’s size varies, forming pull-apart basins and valleys through oblique strike-slip motions. Compressional features result from right shifts in fault strands, forming transverse structural saddles^4^. Asymmetry appears on various scales due to normal faulting, isostatic uplift, and existing topography. Seismically active, DST experiences earthquakes primarily at depths of 20–30 km. Structurally divided into northern and southern segments, the DST's southern part contains continuous valleys controlled by longitudinal en-echelon faults, including the actively subsiding Gulf of Elat^4,15,16^.

The Dead Sea Basin, one of the world's largest pull-apart basins, stretches approximately 150 km from central Arava to the north of Jericho, reaching a depth of 418 m below sea level^4,16,17^. Formed between the Arava basin and the Jericho fault 18–15 million years ago, it is divided into northern and southern basins by a buried salt diapir known as the Lisan Peninsula. During the early to middle Miocene, significant depression occurred with simultaneous sedimentation and subsidence, forming the Hazeva Formation. In the Pliocene, the landlocked southern basin deposited thick evaporites, primarily halite, forming the Sodom Formation. Climate changes led to lake formation, and subsequent slow sedimentation resulted in a deep depression with clastic and evaporated deposits. The southern basin is characterized by distinct subbasins with evaporates and salt diapirs formed through compression, faulting, and salt flow^1,2,15,16^. The northern subbasin, deeper and bordered by vertical faults, exhibits symmetrical graben formation. The southern subbasin's unique nature is attributed to overlapping fault propagation, with the idea of lower crustal flow causing subsidence being debated. An E-W cross-section reveals three structural blocks due to E–W and NE–SW extension and lateral motion^1,2,5,15,16^.

The northern Dead Sea Basin has a thinner sedimentary fill (6–8 km) compared to the southern basin, extending into the lower Jordan Valley. Structurally divided into smaller subbasins, separated by saddles and transverse faults, it becomes shallower and narrower towards the north. The Arnon sink, the deepest subbasin, experienced rapid subsidence in the late Pliocene and Pleistocene. The Kalia fault, an active transverse fault, separates the Arnon basin from the joint lake-land Kalia Basin, confirmed by earthquake activity. Subbasins like Jericho and Fazael, bounded by transverse faults, formed due to motion along the central Dead Sea Fault, with converging boundary faults preventing expansion in the north and south directions^2,5,17^.

The northern basin is considered seismically active owing to the two largest areal earthquakes, i.e., the 1927 6.25 ML magnitude earthquake^18^ and the 2004 5.1 magnitude earthquake^19^, which struck in the last 100 years. Based on the analysis made from the shallow subsurface seismic sections of the Jericho fault, it can be said that the Jericho fault is an active fault with horizontal and vertical components of displacement signified by lateral motion along the fault^17,20^. Moreover, approaching the northern reaches of the DST, the deformations and fault activity subsequently increase. As the DST further extends and structurally links with fault strands of the East Anatolian Fault (EAF), these regions are associated with high seismic activities, as observed for the earthquake mentioned above and its aftershocks^3^.

### FEMR models and generation

In the past decade, research on Fracture-Induced Electromagnetic Radiation (FEMR) has advanced as a valuable geophysical tool, determining recent crustal stresses, visualizing stress modifications in tunnels, and serving as a precursor for geohazards like landslides and earthquake forecasting^21–31^. Lab experiments on various materials, such as chalk, rocks, glass, ceramics, granite, etc., have been conducted to understand FEMR responses, correlating them with physical parameters like crack dimensions, velocity, and frequency of crack propagation^32–41^. Notably, experiments focused on low-frequency electromagnetic radiation from rocks and brittle materials, including concrete, Syracuse limestone, Carrara marble, and green Luserna granite, aimed to predict failure and seismic activity by studying electrical emissions as precursors^42–45^.

Rigorous acoustic emission (AE) and FEMR experiments on various materials aided in visualizing the rate of FEMR counts or FEMR activity with the loading conditions and establishing a correlation between the corresponding stress drop and accumulated energies of the different materials^38,46^. These studies revealed that elastic energy accumulated in rock or coal samples was released in various stages of loading in the form of stress drops characteristic of an FEMR pulse. In other words, FEMR peaks were observed in the regions where the stress dropped abruptly. Additionally, the stress drops represent the propagating microcracks generated on the weak surfaces of the brittle materials and propagate due to increased loading conditions.

Recent seismo-electromagnetic studies enable real-time monitoring of fractures, from laboratory to large-scale geological contexts^28–31^. The Fracture Electromagnetic Radiation (FEMR) method detects Earth's fractures by generating electromagnetic radiation from brittle rock bodies stressed in the near-surface crust^26,34,39,40,47^. External stimuli, like deviatoric stresses in active tectonic zones, induce microcrack formation, leading to material rupture. The “Cascade model” and “Pre-slip model” are pertinent earthquake generation theories^48–50^. The Cascade model explains successive subevents, each triggering the next, culminating in the main shock, where the last subevent determines earthquake magnitude. The Pre-slip model posits that failure starts with an aseismic slip, gradually accelerating until the final rupture, leading to the main shock. These insights advance understanding of fracture processes and earthquake dynamics, facilitating real-time monitoring and potential applications in seismic risk assessment and early warning systems^48–50^.

Israeli and Greek scientists proposed two FEMR staging models before actual earthquakes, hereafter the ***fish-shape*** pulse model^32,34^ and ***the time-series*** model^51^, respectively.

#### FEMR nucleation stages: Fish-shape signals model^34^

Extensive studies on rock failure experiments reveal that crack nucleation and its propagation are composed of the following phases^32,39,52,53^:

Stage 1 (early nucleation stage, Table 1): At the onset of shear stresses reaching critical levels and initiating shear plate displacements, small cracks develop in the weaker material, filling the gaps between corresponding asperity couples. These minute cracks release seismic and electromagnetic radiation (FEMR) at frequencies in the tens of MHz range. Unlike seismic radiation, FEMR penetrates through the medium and exhibits a distinctive fish-like pattern. Subsequent movements of rock blocks result in further micro-cracking in the in-fillings, generating FEMR in the high MHz range. In cases where in-fillings are absent, micro-cracking occurs in the rock blocks.

**Table 1:** A summary of similarity and dissimilarity of FEMR nucleation according to Israeli (Model 1^34^)and Greece (Model 2^51^) research groups.

Model I (stages)	Nucleation stages			
	NSS1	NSS2	NSS3	
Crack type	Compression	Tension	Shear	
Fracturing in	infilling	infilling	asperities	
FEMR frequency	MHz	MHz	kHz	

Model II (stages)	Nucleation stages			Failure/EQ
	1st stage	2nd stage	3rd stage	4th stage
Fracturing in	Heterogeneous “Preparation” zone	Heterogeneous “preparation” zone close to the fault/infilling	Asperities	
Characteristics (FEMR frequency)	Critical (MHz)	Symmetry breaking (MHz)/Tricritical (MHz/kHz)	Extreme event (kHz)	Absence

Stage 2 (medium nucleation stage) (if it occurs): When the perpendicular compressive stress remains below a specific threshold, and the asperities exhibit high breaking strength, it becomes "easier" for the plates to dilate rather than break these asperities. Consequently, the material filling the gaps between the plates is fractured due to tension. The resulting FEMR continues to be in the MHz range.

Stage 3 (advanced nucleation stage): With further increases in shear stress, asperities are broken. The previous MHz range FEMRs are replaced by radiations within the tens to hundreds of kHz range, still displaying the same distinct fish-like shape emanating from cracks oriented parallel to the plates.

#### The time-series model

This model is based on results from the analysis of field observations of MHz-kHz FEMR by multidisciplinary time-series analysis tools. The following four-stage model (Table 1) of the EQ preparation process through FEMR was proposed^51,54–59^:

Stage 1 is defined by observing an MHz anomaly marked by critical dynamics^56,58,60–63^, along with the proposal of a truncated Lévy walk-type mechanism that can bring organization to the heterogeneous system towards criticality^56,64^. It's worth noting that foreshock seismic activity around the epicenter also exhibits behavior characteristic of a critical phenomenon^61,62,65^.

Stage 2—A MHz anomaly marked by symmetry breaking^55,60^ and/or an MHz/kHz anomaly displaying tricritical features are observed^54^. These anomalies originate from either a fracture in the heterogeneous fault "preparation" zone near the fault (resulting in MHz FEMR) or the rupture of infilling material between the two fault sides. This rupture leads to MHz FEMR or sporadic short-duration kHz FEMR observed right after the cessation of the critical MHz FEMR phase, just before the onset of the intense avalanche-like kHz emission^54,59,62,63^.

Phase 3 involves a significant avalanche-like kHz anomaly attributed to the asperities’ fracture. This kHz anomaly exhibits several characteristics of an extreme event, including heightened organization and information content, reduced complexity, strong persistence, and a preferred direction of fracture activities. Additionally, it displays indications of a first-order phase transition^54,58^.

The 4th stage (Table 1) is marked by FEMR quietness across all frequency bands, which is believed to be linked to the preparatory phase of dynamic slip^51,57,58,66,67^.

Both the “Cascade model” and “Pre-slip model” exhibit fundamental consistency with each other and with the global shear failure rock mechanics model^48,49^. In short time gaps between earthquakes, weaker in-filling materials trigger nucleation, forming a heterogeneous “preparation zone.” In contrast, for substantial time intervals between earthquakes, stronger in-filling materials lead to the filling of gaps between blocks, forming the “preparation zone” around the actual fault. Nucleation stages in both models align with Reches & Lockner's model^53^, which posits that cracks form stochastically and exponentially over time, determining precise locations for subsequent shear events^68^.

The fundamental distinction between the basis of both models lies in their approach to FEMR data observation: wide-band measurements are employed in the “fish-shape” model, whereas narrow-band measurements are utilized in the “time series” model. Wide-band measurements provide the means to ascertain FEMR parameters and enable the definition of cracks’ size.

The FEMR parameters and their variation with time before an earthquake are exhibited in this article in the subsequent sections, which will also aim to interpret their corresponding trends.

**Figure 2:**
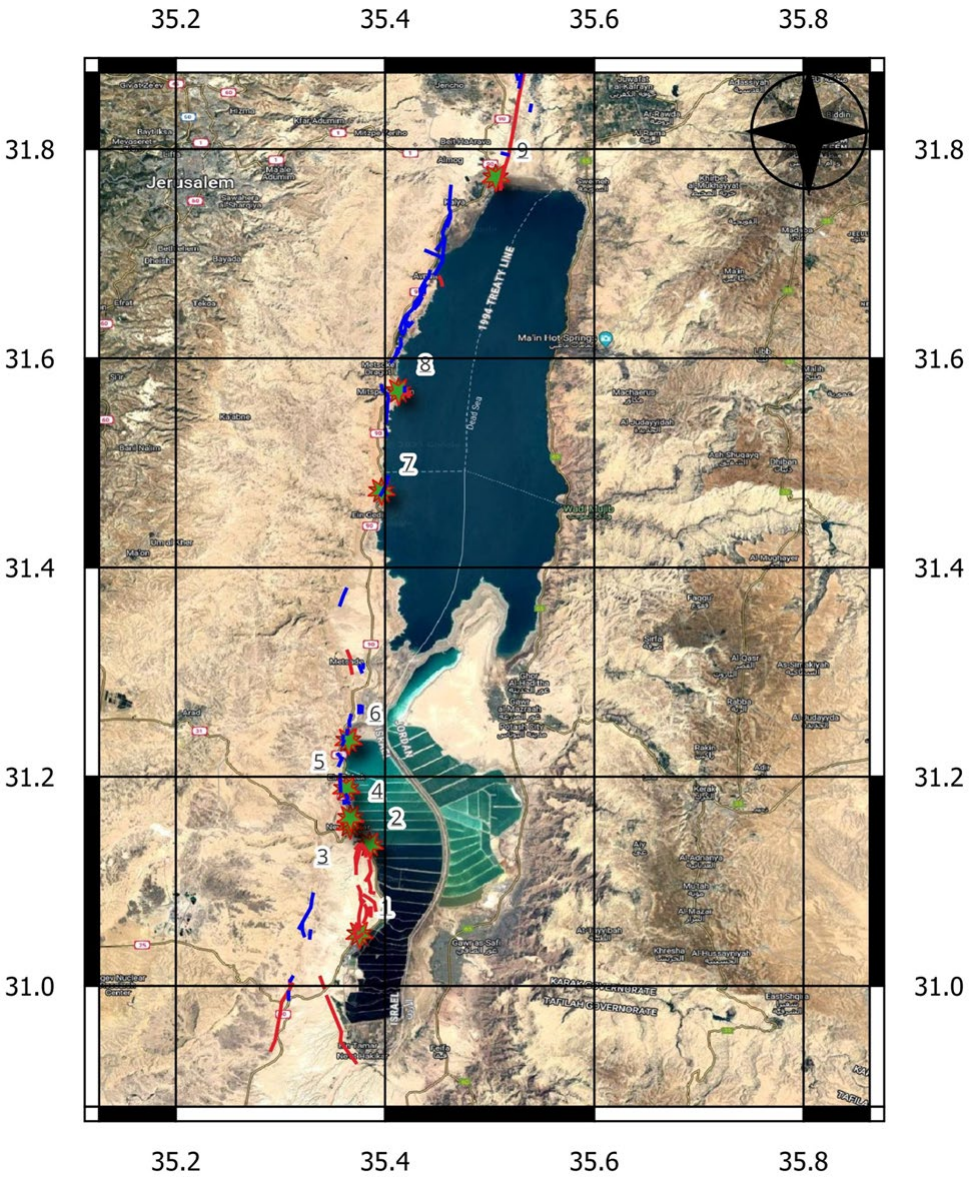
The map of the Dead Sea basin with the superimposed location of FEMR monitoring stations (green stars and numbers). The red and blue lines indicate the location of active and potentially active faults, respectively^82^. This has been modified from Avraham et al.^4^ and a map of active and potentially active faults in Israel provided by the Geological Survey of Israel^82^. The base map is obtained from Google Earth software (https://www.google.com/earth/).

The study was conducted on 20th of Feb 2023 along 9 locations of the DST, with the southernmost location in the Sodom region and the northernmost location in the vicinity of the active Jericho fault (Fig. 2). The day of the field campaign causally coincided with the aftershock event (M_w_ = 6.3) near the Hatay Province at 17:04:29 (UTC) or 19:04:29 Central European Time (CET)^9^.

Determining the locations of the monitoring points roughly spans the entire Dead Sea Basin from the South (Sodom) to the North (Jericho) in the vicinity of main active and potentially active faults. Please refer to Fig. 2, which displays the locations of the monitoring points (1–9) situated in the domain of the blue and red lines indicating potentially active and active faults, respectively.

## Methods

### Instrumentation

**Figure 3:**
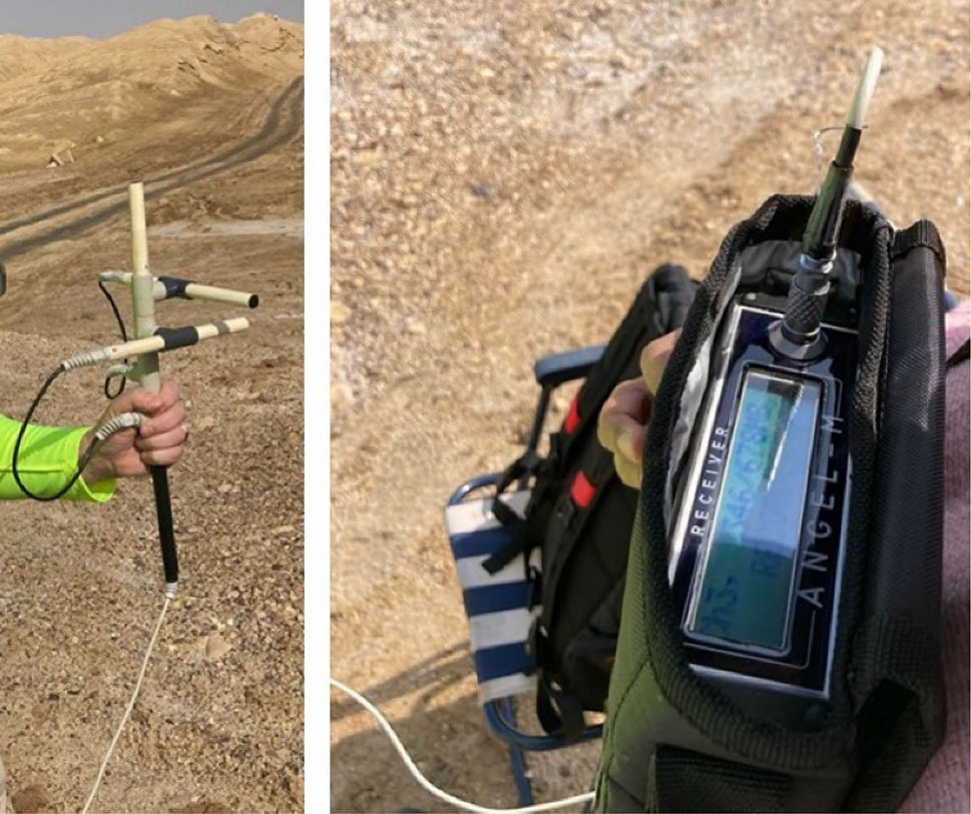
The photographs of the 3D antenna and Angel-M instrument during measurements at the Dead Sea region.

The FEMR data is collected with a portable instrument, ANGEL-M, manufactured by JSC, VNIMI, Russia^24–26,47^. This instrument enables the acquisition of electromagnetic radiation caused by rock fracturing on the geophysical scale^24,25,47,50^ as well as in underground conditions (like mining, tunneling, etc.)^26^. It consists of three pluggable ferrite antennas, a microprocessor-controlled receiver unit, an analog and digital processor unit, an analog–digital converter, and a power supply unit along with a few other essential components such as a RAM and interface card to store the raw data (Fig. 3). The receiver’s front panel contains an LCD and a 10-pin multi-functional connector designed to connect the receiver to a PC, antenna, or charger. The instrument can measure a range of frequencies between 5 and 150 kHz and has a sensitivity of 1 mV/m. At each location, the three antennas are oriented in a mutually orthogonal direction to each other, and measurements are usually taken for 10 s.

**Figure 4:**
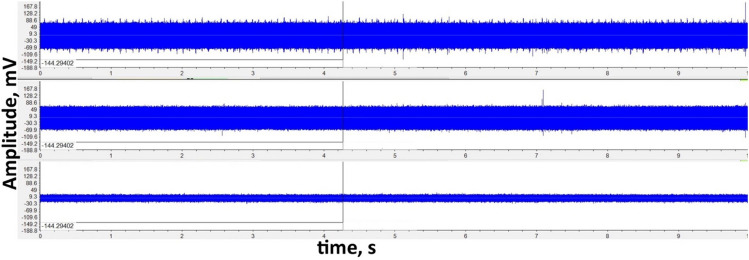
An example of raw data of FEMR observation in three orthogonal channels (Fig. 2—left). The upper record—antenna was directed to the West, the middle record—antenna was referred to the North, and the bottom record—antenna was directed to the Up.

In Frid et al.^69^, the relation of the frequency range, sensitivity/intensity, and activity of FEMR with the features of microcracking was categorically demonstrated. The instrument can record natural and anthropogenic frequencies in a typical field measurement (Fig. 4). Hence, it is required to process the data before its interpretation to eliminate the anthropogenic interferences.

Ensuring the authenticity of the monitoring data is challenging since anthropogenic obscurities often accompany the geogenic FEMR data. Hence, while conducting the surveys, the monitoring locations are chosen in such a way that they are at a considerable distance away from electricity-carrying sources such as electric cables or electric towers, which add to the anthropogenic disturbances in the FEMR readings, which, in turn, causes erroneous results.

### Data filtering and signal processing

Das et al.^24^ have demonstrated the use of ANGEL-M with one antenna procuring data in various geological applications with different modes such as horizontal, linear, and cross-sectional modes^24–26,47^. The current study, however, has used an upgraded version of the instrument, enabling it to perform and visualize 3-D measurements using three antennas simultaneously^50^. The instrument features a 2nd order high pass analog filter to enhance recorded signals and optimize the signal–noise ratio of incoming FEMR signals. Previous studies have successfully distinguished between geogenic and anthropogenic signals based on significant differences in amplitudes and patterns of pulses. FEMR pulses, originating from brittle rock surfaces in the Earth’s crust, have amplitudes in the microvolt range (typically a few microvolts to 20 µvolts), while anthropogenic pulses, artificial sources, exhibit amplitudes several orders of magnitude higher (often in the range of a few hundred microvolts) with a sinusoidal pattern. The instrument, with a “Set Trigger” option, can isolate geogenic pulses within a defined time interval, assessing average pulse amplitude (“A”) and the rate of amplitude growth above the threshold (“B”). In this way, the instrument denoises the FEMR signals from anthropogenic sources^24–26^.

After collecting the raw data from the 9 locations along the Dead Sea Transform (DST), data filtering was employed to devoid anthropogenic obscurities. The procedure is first done by loading the data onto the ANGEL-WORKS software^50^. Figure 4 shows raw data of FEMR records observed in three orthogonal channels. As can be seen, the signal-to-noise ratio is relatively low, and pulse study is impossible without additional processing.

The denoising procedure consists of three filters:

- The “noise removal” filter based on analysis of the noise spectrum; this filter improves the signal/noise ratio of the channel data to quite a considerable extent;
- The “biquad notch filter” is used to adjust the frequency range manually; the range of the noise from different parts of the records was selected for analysis to choose the frequency range to apply the filter adequately;
- The Chebyshev filter is also used to adjust the frequency range manually. In our case, we correct the frequency range between 5 and 50 kHz.

**Figure 5:**
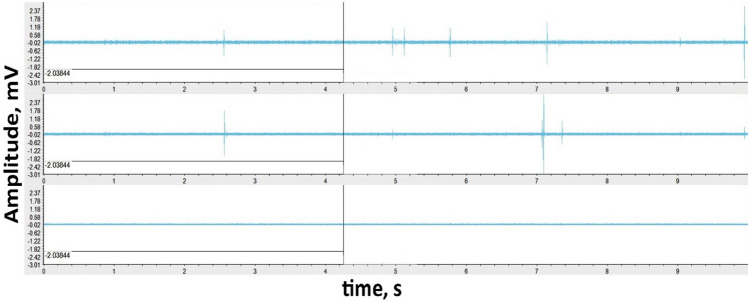
An example of filtered FEMR data in three orthogonal channels (Fig. 2—left). The upper record—antenna was directed to the West, the middle record—antenna was referred to the North, and the bottom record—antenna was directed vertically up. An example of an individual FEMR pulse is shown in Fig. 6.

The filters above vastly improve the signal-to-noise ratio in all three channels, and the FEMR peaks are readily distinguishable (See Fig. 5), which shows results in visualizing the signal amplitude (μV) versus time (10 s) obtained from the three channels simultaneously. This comparison can be seen in Figs. 4 and 5, which show the difference between the raw and filtered data, as analyzed by the ANGEL-WORKS software, especially regarding the difference in the amplitude ranges between them. In most cases, it is most commonly observed that the West and North channels demonstrated a higher activity (pulse/10 s) than the vertical channel, which is primarily dormant or has a meager S/N ratio. However, there are a few locations where all three channels are highly active and have a high activity of FEMR signals for every channel (e.g., Loc 6, 7—Fig. 2). The filtered data for the three channels are saved and exported for further signal processing. Signal processing of the FEMR pulses is done where each FEMR pulse is analyzed, and its corresponding signal parameters are calculated.

A preliminary study has already been conducted in the Eilat region, and the methodology “chain” in acquiring and filtering the data has been tested and validated^50^. The laboratory study also verified the methodology by conducting precise experiments to validate the theoretical concepts guiding the generation and propagation of FEMR signals and determine the shape of a typical FEMR pulse^34^. Several parameters, such as amplitude, frequency, and time of the FEMR signals through different materials, were studied, and the directionality of the FEMR signals from fractures has also been adequately conceptualized and observed through experiments^33,40,46^. Also, various theoretical calculations and experiments have been designed to calculate the approximate attenuation ranges of the FEMR pulses through different materials^33,40^. In addition to this survey, the technique of FEMR has been exploited for various other geophysical applications, such as assessing the modification of stress within a tunnel lying in the Darjeeling-Sikkim Himalayas^26^ as well as delineating landslide-prone slip planes in Central India using the same portable instrument^25^. Although the software ANGEL WORKS was not available for filtering the data from these surveys above, the high-pass analog filter adjusted in the instrument was quite adept in filtering the anthropogenic signals to a large extent.

The major limitation of this study was obtaining data from monitoring points that were directly below intense power stations with a dense network of power lines, mainly in the Sodom region. This caused the corresponding data in the region to be heavily masked with anthropogenic obscurities despite having a 2nd order high pass analog filter in the instrument. Moreover, analyzing this data in the ANGEL-WORKS software and applying the various filters were also inadequate for discerning each FEMR signal from the raw data in any active channel. This resulted in redoing the survey for that particular monitoring point (1–2). Hence, acquiring the data from a few 100 m from the vicinity of the electric lines is desirable to avoid discrepancies in the results. Another limitation of the study is that it might be obscured with very low frequencies (VLF) from man-made sources, and the data might appear to be fracture-induced, emanating from near-surface. This is mainly due to the similarity in the amplitude of the signals, however, the processing of the data in the ANGEL-WORKS software reveals the true nature of the signals. Thus, it is crucial to conduct a thorough geological field survey and ground truthing before conducting FEMR surveys to get baseline data about the geological features and formations in the area, which, in turn, aids in comprehending the subsurface conditions. Additionally, this aids in creating detailed geological maps to visualize the structural features and understand the lithology of the area. The selection of the locations of the monitoring points is subsequently optimized for a comprehensive study.

### FEMR parameters under investigation

The parameters under investigation are divided into groups: the general parameters of FEMR parameters (section “General parameters of FEMR records”) and the parameters of individual FEMR pulses (section “Parameters of individual FEMR pulses”).

#### General parameters of FEMR records

Two parameters of FEMR records were considered for the analysis:

- FEMR activity (FEMR hits hereafter) is defined as the number of FEMR pulses per unit time (in our case, 10 s) recorded for each location, namely the radiation rate.
- The total/cumulative amplitude of FEMR pulses was measured. At the same time, the time axis originated at the EQ occurrence. It extended towards the start of the measurements, precisely the time of observations at location 9 to the time of measurements at location 1. This parameter is analogous to the “Benioff strain release” diagrams”^41,70–72^. Note that it demarcates an “accelerated fracture release (AFR),” increasing as an inverse power-law of time before a macro failure and, in turn, aids in visualizing the continuous development of the upscaling fracture processes in its various stages through time^70–73^. Based on a review of many results, it can be verified that Benioff Strain release, albeit a parameter that cannot be physically measured by the instrument or satellites, can be linked to rock failure observed in the laboratory^74^. It constitutes an essential connection to “critical point systems” and can be used as an accurate proxy for events of macro failure^41,70–72^.

#### Parameters of individual FEMR pulses

**Figure 6:**
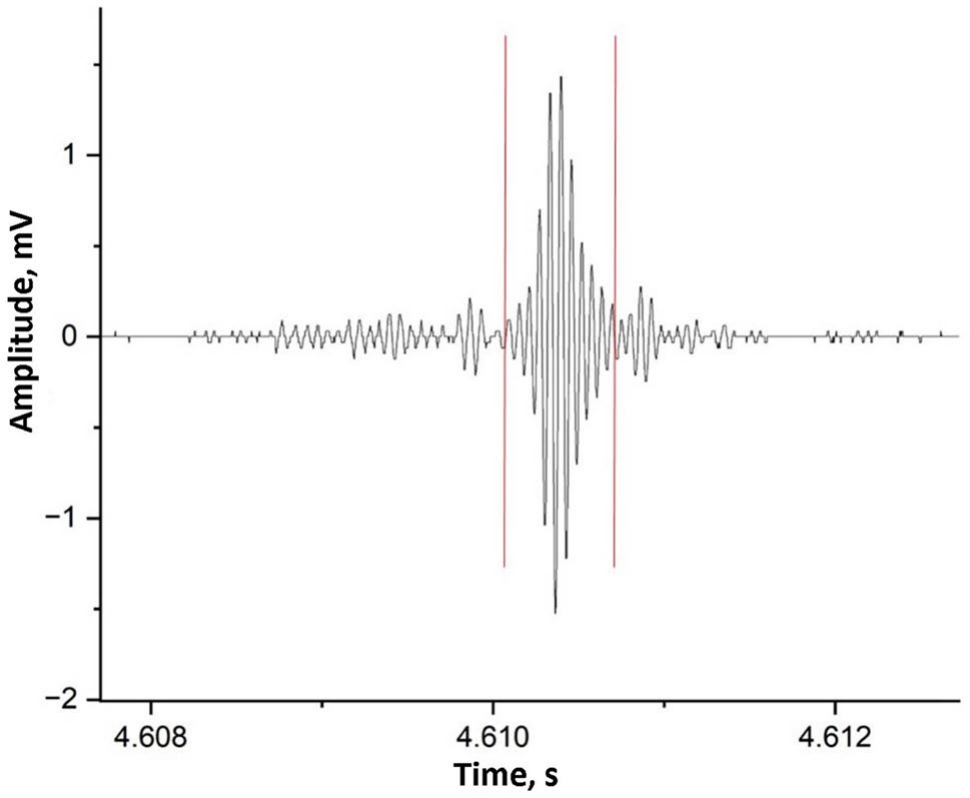
An example of an individual FEMR pulse (zoom-in) emphasized by two vertical red lines.

The amplitude of the FEMR pulse:

A typical fish-like shape of a FEMR pulse obtained from one of the channels is displayed in Fig. 6). Decades of rigorous studies on the origin and characteristics of FEMR pulses have revealed that the pulse shape is invariant to the material type and its loading mode^32–34,36,37,39,40,52^. Furthermore, it is independent of the crack scale, causing the geogenic radiations to emanate. Following the concept of the “Surface Vibrational Oscillating Waves” model (SVOW), where lines of oscillating dipoles caused by the propagating cracks on either of its sides exponentially decay into the bulk of the material are analogous to that of Surface waves (or Rayleigh waves)^32,34^ Hence, field amplitude (A) can be quantified as follows:

1 $$A = A_{0 } \times e^{ - \alpha R}$$

where A_0_ is the source amplitude, α is the attenuation factor, and R is the distance between the source and the measuring antenna^50^. Kindly note here that since we consider the source of the signals emanating from the brittle rock surface of the upper crust of the field area, it is assumed to be approximately 5 km from the measuring antenna^33,39,40^.

The field amplitude parameter (A) is dependent on the gain of the measuring instrument (G) as follows:

2 $$G = \frac{A}{{A_{OUT} }}$$

where A_OUT_ is the signal amplitude incorporating the “Antenna Factor (A_F_)” which can be expressed as:

3 $$A_{F } = 20log\frac{{E_{{ANT_{IN} }} }}{{V_{OUT} }}$$

Here, *E*_*ANT_IN*_ is the amplitude of the input signal in the antenna or the incident electromagnetic field, and V_OUT_ is the output voltage from the antenna. The gain of the instrument and, by that extension, *A*_*F*_ are considered to be constant due to the frequency bandwidth limitation of the device.

c. The Rise Time: the time difference between the start of the pulse and its maximum is termed “Rise Time” or *T′*. It was computed for every pulse for all the channels in every location.

d. The pulse energy: In the realm of signal processing, the energy of a continuous time-dependent signal *A(t)* can be considered to be the area under the square of a time-dependent function or, in other words:

4 $$E_{x } = \mathop \smallint \limits_{0}^{T} \left| {A\left( t \right)} \right|dt$$

Since the units of FEMR amplitude after antenna factor correction are V/m, the energy units are V^2^s/m^2^.

Where T is the pulse duration, in our case, this value equals the sum of rise and fall times. Namely, Ex = *E*_*RFT*_ is a measure of signal strength.

As a result of rock compression, the amplitude of the signals is the square root of the FEMR energy, which can be linked to the fractal nature of processes controlling earthquakes^70,72^. Moreover, it was shown that crack-inducing FEMR increases the pulse amplitude. Hence, it can be assumed that a more accurate calculation can be using not the entire pulse duration *T* but the time to the pulse peak *T’*, namely, the pulse rise time:

5 $$E_{RT} = 0.5A^{2} T^{\prime}\sim A^{2} T^{\prime}$$

And its unit will be V^2^s/m^2^.

e. The crack length: A correlation between T′ and crack length (*l*) can be made based on the SVOW model^32,34^, where it can be seen that an increase in the FEMR pulse amplitude is dependent on the growth of the crack. Subsequently, on halting the crack formation, the pulse amplitude also starts to decay, yielding^32,34^:

6 $$T^{\prime} = \frac{l}{{v_{cr} }}$$

where v_cr_ is the crack speed.

f. The crack width: According to the SVOW model, crack propagation is limited by the crack width (*b*) due to restrictive movements on either side of the propagating crack. Hence, b is given by:

7 $$b \cong \frac{{v_{R} }}{2f}$$

where *v*_*R*_ is the Rayleigh surface wave velocity, and *f* is the frequency.

g. The crack area:

From Eqs. (6) and (8), we hence get

8 $$\frac{T}{2f} = \frac{l \times b}{{v_{R} v_{cr} }}$$

where (CA) ~ *l* × *b* is the crack area. The above relations were also verified experimentally for various materials such as chalk, granite, ceramics, PMMA, marble, etc^32–34,36–38,46^.

## Results

**Figure 7:**
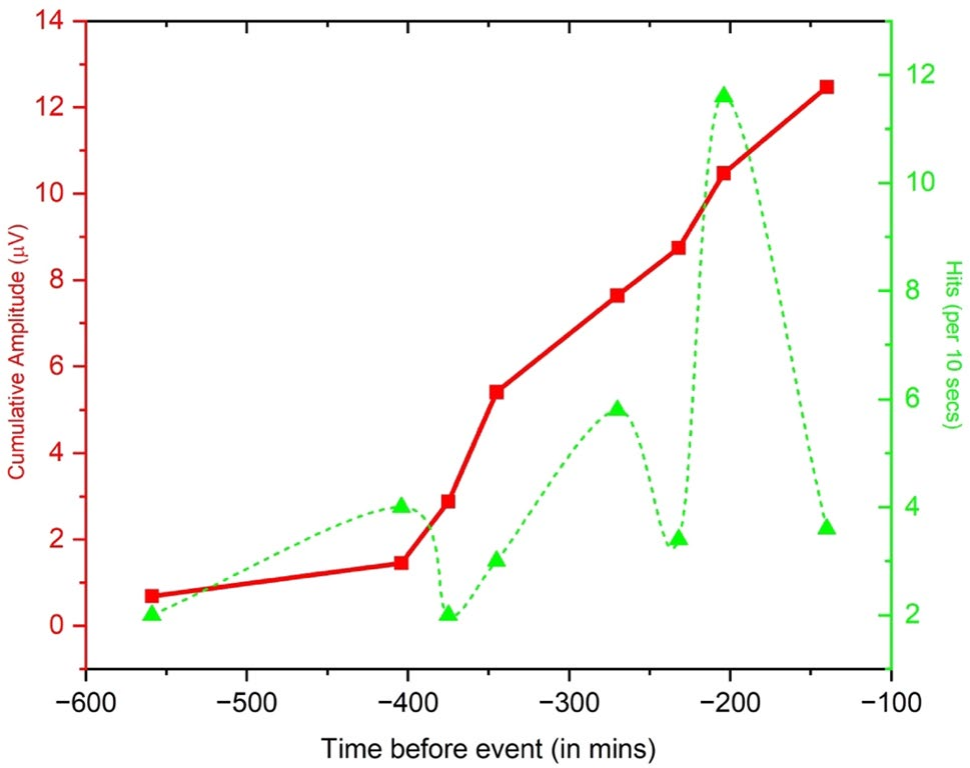
The chart of cumulative FEMR amplitude (the “Benioff strain release diagram” TDBS)—red curve and FEMR Hits (section “General parameters of FEMR records” for definitions and details)—green curve versus the time before the earthquake event.

Figure 7 shows the chart of cumulative FEMR amplitude (the time-dependent “Benioff strain release diagram” or TDBS) and FEMR Hits (section “General parameters of FEMR records” for definitions and details) versus the time before the earthquake event. The time axis represents the cumulative time difference between the moment of data recording at a specific station and the time when the earthquake occurred. Here, three distinct stages are observed. The first stage starts around 560 min before the earthquake event and continues until about 400 min before the event. Here, the slope of the TDBS graph is increasing very gradually. The second stage, which is between 400 and 230 min before the event, demarcates a steep rise in the slope of the TDBS graph. Subsequently, to this point (− 230 min), the third stage of the Benioff strain starts, where the plot increases steadily, and the differential change in the values of cumulative amplitude is much less.

Two trends are seen here for the FEMR Hits: (1) It has an increasing trend with *position*, i.e., the activity of the channels continuously increases as was recorded from the southernmost location (1) to the northernmost location (9) (Fig. 2).

(2) The activity also increases constantly as it approaches the *time* of the earthquake event. The difference between the two parameters lies in the Benioff strain release plot, which was constructed by summing the amplitudes at each location and accumulating these sum values. Specifically, the Benioff value for the first location is calculated as the sum of amplitudes at that location. The second location is computed as the cumulative sum of the first and second locations, and so on. At the same time, FEMR hits are a time-dependent parameter representing each location's FEMR activity (See section “General parameters of FEMR records”). Hence, from Fig. 7, these two parameters do not have a direct one-to-one correlation, suggesting a possible complex dependence. A plausible correlation can be made concerning the second-order differential of the Benioff Strain with FEMR hits. The second peak of hits forms from approximately − 400 min, reaches its second peak around − 270 min, and then starts to decay. Here, the second-order differential change in the second peak of the hits corresponds to the second stage of the Benioff strain plot, where it has a steady slope or in other words, almost no change in its slope. This might be ascribed to the decay of the hits from its second peak onwards. The 3rd peak of the hits starts to form from approximately − 235 min and reaches its peak around − 200 min and, after that, begins to decay. In this 3rd peak of hits, the value is the highest compared to its entire range. Its corresponding point in the Benioff strain has also increased significantly compared to its previous stages, which can be attributed to its sudden increase in the slope or a positive second-order differential value. Just after the 3rd peak point of the hits, its slope starts to decay. In the corresponding Benioff strain graph, the increase in the cumulative value of the amplitude is slower, i.e., the 2nd-order differential is negative. Hence, here, the 1st order change is positive, but the second-order change is negative, which might result in the activity falling off.

**Figure 8:**
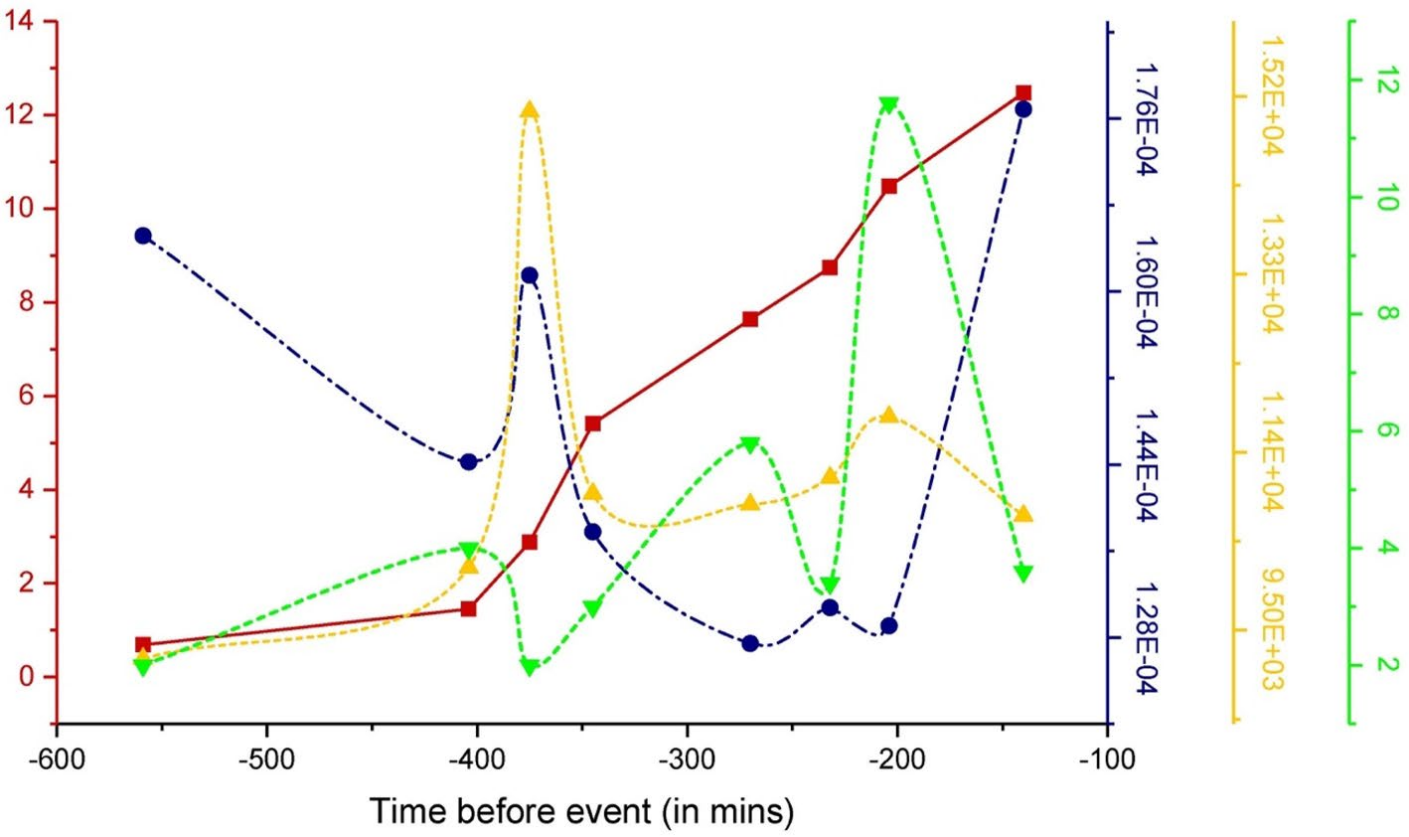
The plots for variation of the rise time (T′)—blue, frequencies (*f*)—yellow, FEMR hits—green, and the cumulative FEMR amplitude (Benioff strain release) versus the time before the earthquake.

Figure 8 shows the plots for variation of the rise time (T′)—blue, frequencies (*f*)—yellow, FEMR hits—green, and the cumulative FEMR amplitude (Benioff strain release)—red curve for the different locations versus the time before the earthquake.

Note that the antenna factor G = 18,000 as per the instrument specifications; hence, A (Eq. 2), as observed from the obtained results, is in the range of 0.6–2 μV. Thus, A_OUT_ ≅ (3–11) × 10^−11^ V and the frequencies of the received signals (*f*) range from 9 to 15 kHz. On the other hand, the rise time (T′) computed for individual pulses varies from 0.12 to 0.18 ms.

In general, there is a decrease in the T′ trend except for two points in the graph where it peaks. The first peak is reached around 370 min before the earthquake, where the plot steadily increases and then decays. Correlating this with the Benioff strain plot, this point of the first T′ peak occurs in the beginning second stage of the Benioff strain, and as the former parameter keeps increasing gradually in its slope in its second stage, T′ keeps decreasing. This is also the juncture where the first cyclic period of the FEMR hits damp down, and the second, more intense cyclic period commences. The most noteworthy juncture is the 3rd stage of the Benioff strain plot, where around 200 min before the event, a tentative “saturation junction” is obtained for the T′ plot, where its value increases anomalously after this point. In the 3rd stage of the Benioff strain, where its values are higher, the general trend of T′ diverges from its path and anomalously increases. On the other hand, the frequency plot (*f*) has an increasing trend, especially in the second stage of the Benioff plot, where its peak corresponds to the peak of *T*′. Following this point, its values steadily dip down and then increase gradually, corresponding to the slower increase in the values of the Benioff strain. Following the “saturation juncture”, its values decrease, contrary to the abnormal growth in the T′ plot. Considering the FEMR hits at around 230 min before the earthquake, its values increase abruptly in the 3rd stage of the Benioff plot, maintaining its cyclic periodicity with a growing trend. This frequency correlation from − 230 min onwards is more apparent concerning the FEMR hits.

**Figure 9:**
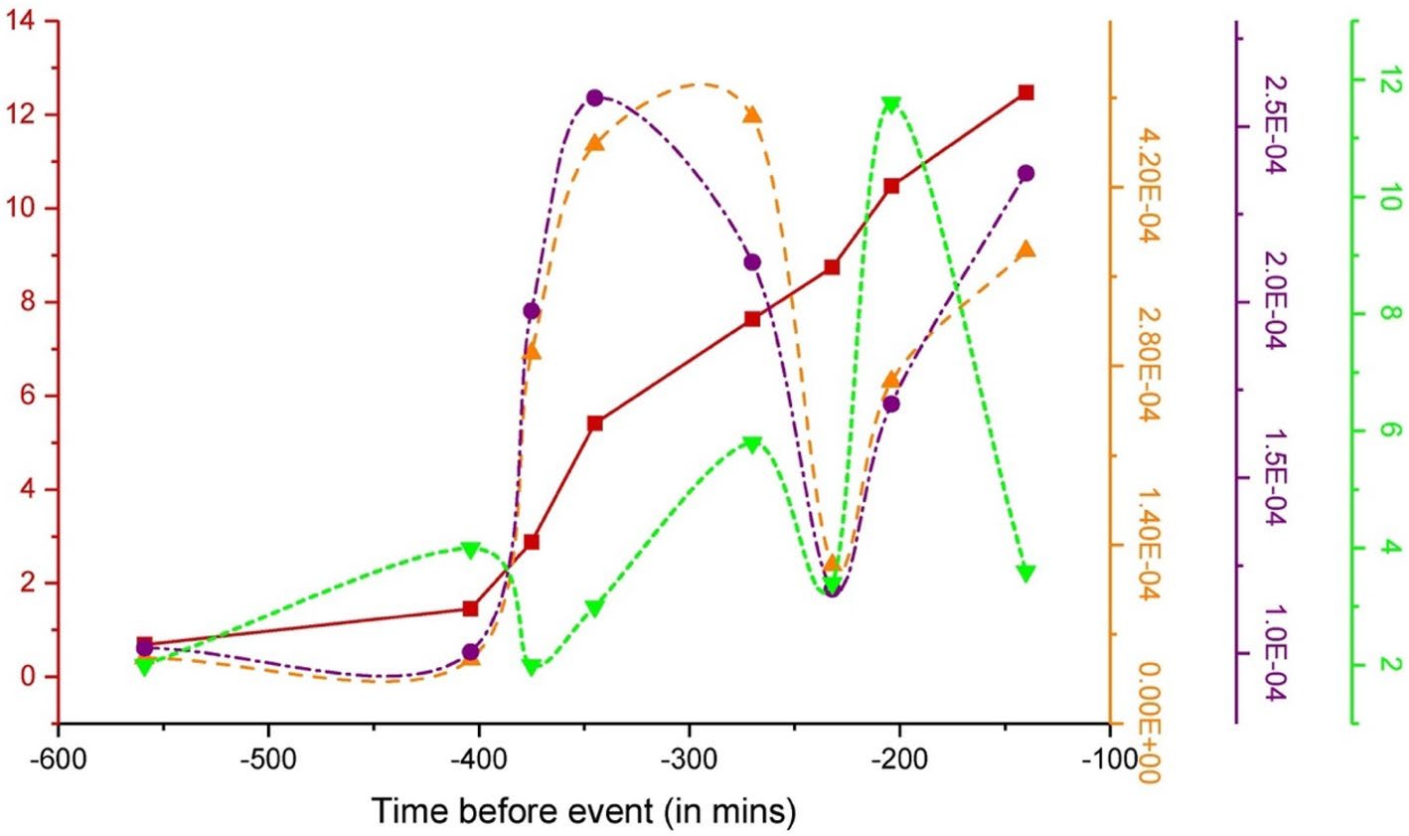
The plot for variation of energy parameters $E_{RFT}$—purple curve $E_{RT}$—orange line calculated based on Eqs. (4) and (5), respectively. FEMR hits—green plot, and the cumulative FEMR amplitude (Benioff strain release)—red curve for the different locations versus the time before the earthquake.

Figure 9 shows the plot for pulse energy variation calculated by two methods based on Eq. (4) (${E}_{RFT}$—the purple curve, and Eq. (5) (${E}_{RT}$—the orange curve), respectively. In addition, Fig. 9 shows the FEMR hits chart—the green line, and the cumulative FEMR amplitude (Benioff strain release)—the red curve for the different locations versus the time before the earthquake. Analysis of both energy charts shows that the ${E}_{RFT}$ value ranges 1–3*10^–4^ (μV)^2^ s/m^2^ while the ${E}_{RT}$ value ranges 0.5–3.7*10^–4^ (μV)^2^ s/m^2^. It can be seen (Fig. 9) that the ${E}_{RFT}$ and ${E}_{RT}$ values correlate pretty well, thus validating the obtained results. These parameters' values start to increase in the second stage of the Benioff strain release chart, correlating with the increase in FEMR hits. The subsequent dip down of both parameters can be noted precisely at the end of the second stage of the Benioff strain release curve and FEMR hits. Following this, at the start of the 3rd stage of the Benioff strain release and FEMR hits, the value of both energy parameters ${E}_{RFT}$ and ${E}_{RT}$ ascends steeply again. This complex compounded plot of calculated parameters displays a Full-width half maximum (FWHM) in precisely the second stage of the Benioff strain release, and FEMR hits plots, which supports the statement above of the two essential junctures or trend markers: one starting around 400 min and the other one which is about 230 min before the earthquake event. It is also consistent with the earlier trends of other parameters.

Therefore, it can be said that the energy parameters calculated concerning Eqs. (4) and (5) are not just statistical proof but rather more physical evidence of how the energy of a FEMR signal is expected to evolve before an event of macro failure. In the 2nd stage of the Benioff strain release plot, the energies are the maximum, although the recorded FEMR hits are not the maximum in this stage. The energies are proportional to the square of the amplitude of each signal and the number of signals. Therefore, the maximum accumulated energies observed in the second stage, even with a lower rate of FEMR activity (see section “Parameters of individual FEMR pulses” for definition) compared to the third cyclic stage of hits, suggests a higher amplitude of each signal in this stage.

**Figure 10:**
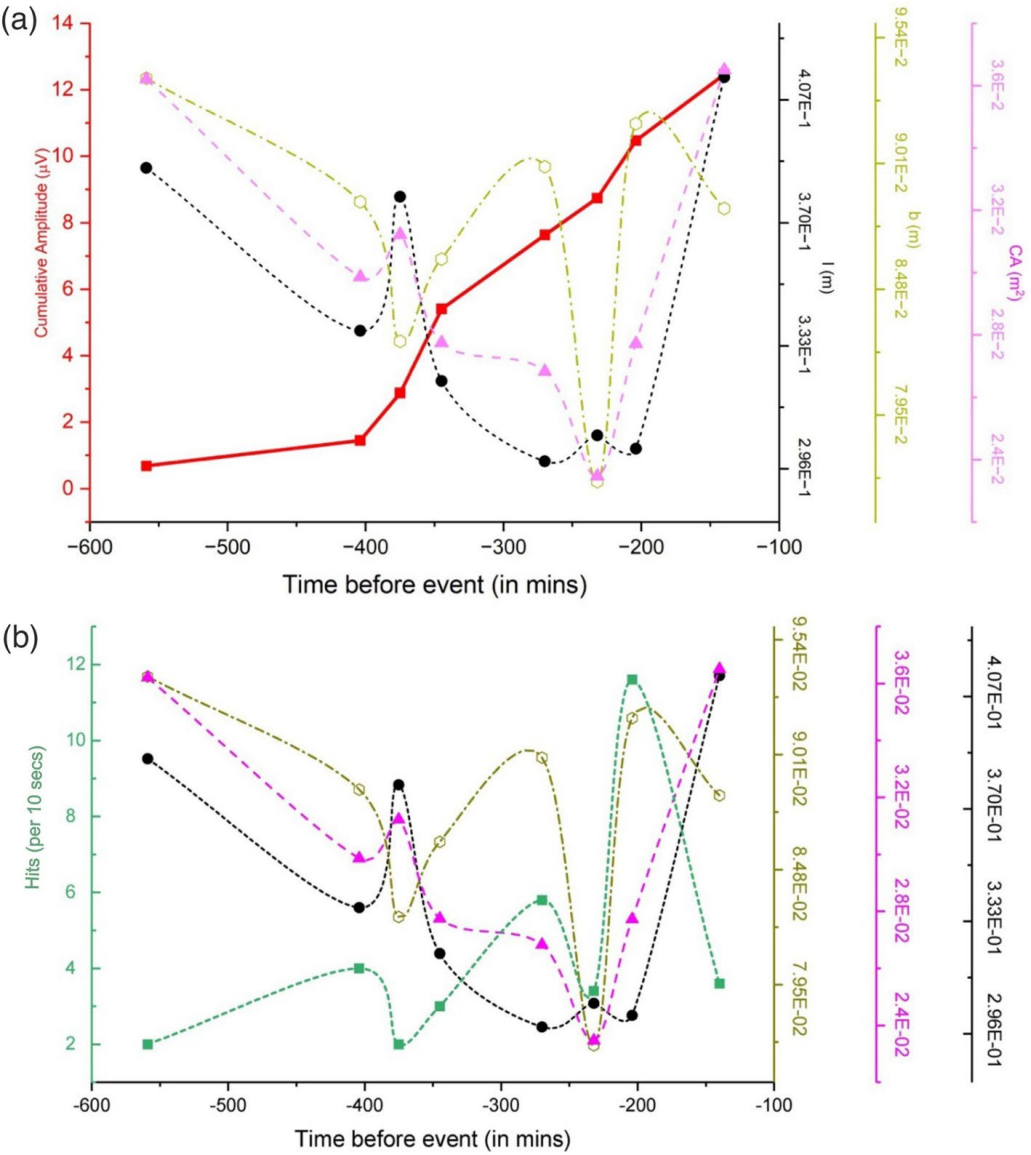
(**a**) The plot for crack length (black line, m), crack width (olive line, m), crack area (purple line, m^2^), and the cumulative FEMR amplitude (Benioff strain release)—the red curve for the different locations versus the time before the earthquake, and (**b**) the aforementioned parameters and FEMR hits (the green line).

Figure 10 shows the plot for crack length (black line), crack width (olive line), crack area (purple line), and the cumulative FEMR amplitude (Benioff strain release)—the red curve versus the time before the earthquake Fig. 10a and the FEMR hits chart—the green line (Fig. 10b).

Projecting the calculation of the individual pulse parameters to the crack dimensions, v_R_ and v_cr_ are considered to be 2600 m/s and 2340 m/s, respectively (for our study area)^50^, yielding the values of *l*≈30–41 cm when *T’* varies between 120 and 180 µs and b ≈ 7–9 cms from Eqs. (6) and (7), respectively. Hence, we see from Fig. 10 that* l* follows the same decreasing trend as that of *T′* (as expected from Eq. 6) and peaks around 370 min before the EQ event and anomalously increases in the 3rd stage of the Benioff strain plot (Fig. 10a) and FEMR hits (Fig. 10b). On the other hand, *b* dips down around the above first juncture point at the second stage of the Benioff strain release (Fig. 10a) and FEMT hits (Fig. 10b). It subsequently has a steep decline at the beginning of the 3rd stage of both general FEMR parameters, i.e., around 260 min before the event. A more relevant marker for crack dimensions in this plot comes from crack area (CA), providing a 1st order contribution of parameters *l* and *b*. Hence, the local maximum of CA comes at the aforementioned first juncture point, which steadily decreases and reaches a local minimum at the end of the second stage of both general FEMR parameters. Like the previous parameter, this demarcates a “saturation point” following which its value anomalously increases in the 3rd stage even though the cumulative amplitude increase rate is comparatively less and FEMR activity just before the EQ event is relatively low. An accurate comparison of changes of parameters *l*, *b,* and CA versus the FEMR hits (Fig. 10b) portrays that in the first cycle of the hits, there is a steady decrease in the crack dimensions, while in the second cycle, demarcated by an increase in its peak value compared to its previous cycle, *b* follow the same trend, and *l* and CA continue to decrease. The 3^rd^ stage of the hits cycle shows similar trends for all the crack dimensions in which all the parameters increase in their respective values. More precisely, hits and *b* follow the same trend while *l* and CA increase anomalously.

Table 2 displays the Benioff strain release (BS) stages versus other FEMR parameters and their corresponding plot trends. In summary, in the first stage of the Benioff strain release plot, there is an increase in the FEMR hits and frequency. At the same time, there is a steady decrease in the rise time (T’) and the crack dimensions (*l, b*, CA). The energy parameters have almost no change in their values at this stage. The second stage of the Benioff strain release plot is demarcated by a steady decline in the FEMR hits and crack width, while all the other parameters have an increasing trend. Finally, the 3rd stage of the Benioff strain plot is associated with an increase in the FEMR hits, energies, and crack width, while *T′*, crack length, and CA show an anomalously high rise in their respective trends. Only frequency decays in this stage.

**Table 2:** The Benioff strain release stages vs. FEMR parameters (D, I, SI, AI and NC mean decrease, increase, slightly increase, abnormal increase, and not change, respectively).

Stages	Time	BS	Hits	*T’*	*f*	$E_{RFT}$	$E_{RF}$	*l*	*b*	CA
1	− 600 < T < − 400	I	I	D	I	NC	NC	D	D	D
T12	− 400		D	I	I	I	I	I	D	I
2	− 400 < T < − 230	I	I	D	D	D	D	D	D	D
T23	− 230		D	NC	SI	I	I	NC	I	I
3	− 230 < T < − 150	I	I	AI	D	I	I	AI	I	AI

## Discussion

### Interpretation of the FEMR parameters

Figure 7 displays a plot between the cumulative FEMR amplitude and the time before the earthquake and defines three distinct stages. This also represents smaller stress release processes owing to “crack shielding”^70^. The slope of the first stage (− 600 to − 400 min) is gradual, followed by a steep increase of slope in the second stage (− 400 to − 230 min) and then a steady increase in the slope with significantly less change in the differential evolution of the values of the cumulative amplitudes (− 230 to − 150 min). These could be reminiscent of the stages of nucleation above, where the 1st stage, with its gradual slope increase, represents the early nucleation stage or the quasi-static stage associated with a steady increase in stress levels. Stage 2 of the Benioff strain release diagram represents the “accelerated stage” where the “Process zone” starts to dilate and extend owing to an increase in the loading conditions and an increase in the number of more minor cracks followed by stage 3, which is associated with a “dynamic propagation”. This is related to a state of irreversibility where the gradual and steady slope indicates that with an increase of time, the resistance (provided by the newer opening cracks) to the increasing stress gradually reduces, which would eventually lead to a rupture or macro failure.

Note that the data from the last station (9 in Fig. 2) was recorded approximately 150 min before the earthquake occurred, so we could not represent an actual “saturation” stage^70^ in our Benioff strain release diagram, which would be representative of the earthquake. This would eventually lead to the relatively constant Benioff strain curve with no change in the cumulative amplitude with passing time until an EQ.

Figure 7 also shows the wave-like behavior in FEMR activity (the number of hits per unit of time), where the stages of FEMR excitation (1, 2, 3 in Table 2) are replaced by the periods of relative silence (T12, T23 in Table 2). In addition, each subsequent half-wave of FEMR activity is higher than the previous one. It can be assumed that such wave-like behavior is probably related to the wave-like behavior of stress accumulation and release (stress drop) in a relatively micro-level (fracturing level) during the EQ development. For example, when external stress reaches a specific level, new fracture surfaces emerge due to the opening of fresh cracks; this leads to a peak in FEMR activity. This peak marks the region where stress is released, followed by a subsequent reduction in stress and a decline in FEMR activity (e.g., T12 and T23 in Table 2).

Moreover, the subsequent increase in the half-wave amplitude of FEMR activity (Fig. 7) probably reflects a rock “memory” behavior (Kaiser effect)^75^ when the rock fracturing begins only when the stress level is higher than the previous one. This is consistent with the studies across various brittle materials where the stress drop is the highest before the event of a macro failure^46,76^.

Note the periodical behavior of FEMR energy calculated by two different methods (Fig. 9), when the first maximum was noted at the second stage (T2 in Table 2) about 400–230 min before the EQ followed by the drop in FEMR energy (T23 in Table 2) and the subsequent increase before the EQ (T3). The rise in FEMR energy is attributed to an extension in the time up to the pulse's maximum (*T',* Fig. 8). Consequently, the overall duration of FEMR pulses increases, aligning with findings in a previous study^46^. When comparing FEMR activity and energy variations at stage T3 (150 min before the EQ), it becomes apparent that despite the decrease in FEMR activity, the FEMR energy values continue to rise. This suggests that their energy remains higher even though FEMR pulses are less frequent.

Moreover, it has been proposed that FEMR energy generated due to fracturing in brittle rock bodies is proportional to the charge density accumulated on either side of a propagating crack. In the second stage of the Benioff strain plot, the increase in the number of cracks or, rather, the increase in the surface area of the “Process Zone” will thus increase the system's energy. Now we can see that in T23, the energies damp down steadily. This can be attributed to the dissipation of the surface energy of the cracks in breaking the asperities prevalent in any brittle surface. Overcoming the frictional strength of the asperities leads to a steady dip in the value of energies in T23, which starts to ascend once again in the third stage of the Benioff strain plot. This is because the energy once again emanates from the generation of new crack surfaces in the dense “process zone” until the microcracks coalesce.

The crack length (*l*) in Fig. 10 has a general decreasing trend till the end of the second stage, meaning that an increase in FEMR activity and Energy occurs due to the rise in the number of cracks. However, from T23 (Table 2), it increases anomalously for the 3rd stage of the Benioff strain plot. Now, it is possible to comprehend this bizarre behavior in the final stage of the Benioff strain plot, which can be attributed to the rapid extension of the sheared process zone across the surface of the brittle bodies and coalescence of the microcracks before the final rupturing event. In the first stage of the Benioff strain plot, *l* steadily decreases and ascends towards T12. This could be ascribed to the early nucleation phase, where an intense interaction exists between the close “stepped cracks”. This leads to cracks opening due to tensile stresses or normal stresses of its neighboring cracks and the closing of some cracks. This intense interaction initiates the process zone, which propagates and increases in length.

The other variation of crack dimension observed is that of the crack width (*b*), which has a contrasting trend to that of *l* in the first two stages of the Benioff strain plots, indicating that the cracks become narrower as they propagate through the medium of the study area. As mentioned above, an increase in the crack density leads to a rise in the surface area of the material, and the surface energy generated is a result of the crack area and the specific surface energy. Hence, the energy released by the crack growth or increase in the crack length should suffice for the energy needed for crack propagation, and a better representation of the crack dimension, which encompasses the trends of both *l* and *b* is that of the crack area (CA), which follows a similar trend to *l*.

As seen from Fig. 10, the range of crack sizes observed in the study is as follows: *l* = 0.3–0.4 m and *b* = 0.08–0.1 m. Based on quite similar crack length and width dimensions, they can be assumed to be defined as tensile. This assumption is consistent with the previous studies^77^. For example, fractures produced during the 1968 earthquake at the Coyote Creek fault in California are intensively branched by rapid rupture. The angular behavior of the branching ruptures in eight forks suggests tensile fracturing in that event^77^.

Additional observations that support this implication include a series of distinguished cracks that show openings of 20–30 mm per rupture, the symmetrical and bilateral forking, the high-intensity and angular shapes of individual branches, the opening of grabens associated with several bifurcations, and the patterns of en-echelon fractures which reflect mixed mode at the rupture surface. Hence, contrary to previous interpretations, according to field evidence and fracture mechanic theory, the fault bifurcation and openings along the Coyote Creek fault in 1968 are not compatible with local tension caused by the faulting. More likely, the set of tensile microcracks was formed in a shear compressional regime during an early transitional stage of the earthquake.

Fractures probably occurred by different mechanical modes at depth and the surface. Although faulting may have originated by shear at depth, rupture at the surface was dominated by far-field tension associated with NE–SW extension in South California^77^. Central to the understanding of the formation of stationary tensile microcracks within a shear compressional regime is identifying the transformation between its early tensile nucleation stage and the later growth of fractures in the shear stage. This distinction characterizes various types of transformations in nature. Whether it is a crystalline polymorphic transformation like the one that occurred due to temperature change between hexacelsian (hexagonal) and celsian (monoclinic) having the same chemical composition^78^. Or in earthquake transformations like the one described here, which occurred with time between a zone of small stationary tensile microcracks and a zone of large dynamic fractures, including faults. Intriguingly, previous studies on the transformation between early nucleation to later growth^78^ and the unexpected formation of tensile fracturing in compressed shear regimes^77^ were successfully applied by the FEMR method to the earlier forecast of EQs^40,50,52^.

### Geological applications of the FEMR technique

The FEMR technique has been utilized as a prolific geophysical tool for various geological applications. They are as follows:

Real-Time Earthquake Monitoring: In terms of real-time monitoring, an improvement in the FEMR technique has enabled real-time monitoring of the fracture process not just pertaining to laboratory scale but also projected to large-scale geological studies known as seismic-electromagnetics^51,54–59^.

The primary motivation behind this research section is that owing to the order of magnitude differences in space and time scales between lab-scale and large-scale processes, the electromagnetic anomalies will possibly be revealed in the final stages of earthquake generation in the geological scale of observations. The study deployed telemetric stations in Greece, recording FEMR emissions from 1992 to 1995 and later forming a telemetric network nationwide^28–31,54–65^. The data revealed electromagnetic anomalies before significant earthquakes, with critical features observed through various analytical methods. The study also explores ultralow-frequency electromagnetic precursors and their correlation with earthquake events. Overall, the telemetric monitoring stations contribute to understanding different stages of earthquake generation and establishing a correlation between a region’s seismicity and associated precursory electromagnetic emissions. Telemetric observation stations recorded electromagnetic emissions (EME) before significant regional earthquakes.

For example, before the October 12, 2013 earthquake near the west coast of Chania, Greece (M_w_ = 6.4), MHz EME was detected around five days before the main shock event. Analysis using two independent methods, the “method of critical fluctuations (MCF)” and “natural time method,” revealed critical features indicating that the geophysical processes leading to the earthquake were in a crucial state. The natural time method, applicable even with limited data, showed the “Critical window (CW)” of EME in the MHz range during the same period as the foreshock’s criticality, suggesting a possible correlation between recorded electromagnetic signals and the impending earthquake event. Using the method of critical fluctuations, FEMR signals in the MHz range before earthquakes in Durres (Albania) and Chania (Greece) in November 2019 exhibited critical and tricritical characteristics^54–65^. A tricritical point was observed where first and second-order transition points meet. Initially, both earthquakes showed critical behavior, indicating a crucial state in the system. Subsequently, for the Durres Earthquake, tricritical dynamics in the MHz range suggested an approaching earthquake or progress to a first-order phase transition in the fracture system. In the case of the Chania earthquake, symmetry breaking in the MHz range indicated the achievement of the second-order phase transition. Critical fluctuations were also applied to FEMR emissions before earthquakes in the eastern Aegean Sea region, showing critical conditions and identifying a tricritical crossover in electromagnetic signals preceding the events^60–65^.

In summary, the FEMR features observed before significant aftershock will be used for incorporating FEMR channels in existing seismic nets, allowing hazard monitoring much earlier before its occurrence.

Determination of recent horizontal near-surface stress azimuth: The technique of FEMR has been used to determine the azimuth of recent near-surface horizontal stresses for a broader area in a limited period^24^, comparable with the data obtained from conventional sources. An underlying basement fault can cause the stress azimuth to realign itself with its trajectory and cause the azimuth to vary locally depending upon the strike of the fault escarpment^24^. It has also been used to determine the regions prone to active tectonism by conducting linear FEMR surveys.

Delineating Landslide-prone slip planes: We have used this technique to delineate landslide-prone slip planes atop a hill^25^. This technique can be profitable for delineating precarious slopes by correlating FEMR amplitude and the safety factor. Hence, a zone of instability can be restricted when anomalously high amplitudes of FEMR values are obtained in the regions of weak slip planes due to microcrack/nano crack propagation, whose number increases in abundance in an area on the verge of slope failure. Analyzing the survey results from a hill; we can detect anomalously high FEMR amplitude values for regions where a chunk of soil mass has started to subside and get wasted, or significant cracks have begun forming on the ground. Subsequently, the quantification of the “Factor of Safety” (i.e., a ratio of the resisting forces to the shearing forces) with the FEMR amplitude marked the extent of the instability of those particular regions^25^.

Seismic moment estimation: Frid et al.^79^ suggested a new method to calculate the seismic moment based on electromagnetic radiation emitted during the fracturing stage in their most recent work. This method is quick and easy to implement and can be profitably used for calculating engineering failure intensity by measuring the seismic moment at the nucleation stage of an earthquake. Other co-seismic devices, such as seismographs or seismometers, are ineffective at such a stage. The results of this method for minor fractures arising during the nucleation stages of an earthquake agree with the former laboratory seismic measuring technique. These measurements can then calibrate the seismic devices to estimate the seismic moment during the catastrophic stages of earthquakes when these seismic devices are suitable and no anthropogenic electromagnetic radiation is present.

Forecasting Rockburst Hazards: This technique has helped assess the region of rockburst hazards in coal mines and tunnels. Intense micro fracturing close to mining operations leads to an increase in the likelihood of rockburst. This fracturing causes an increase in the amplitude of the FEMR pulses by almost two orders of magnitude depending upon the mining area. Rigorous surveys have been done in such areas to reveal the change in FEMR pulse amplitude due to “pillar loading” and unloading^80^. Furthermore, Li et al.^46^ conducted experiments on different coal samples to reveal FEMR anomalies with an abrupt drop in stress. A charge distribution model has been proposed to measure charge separation responsible for the anomalies that occur on the newly generated cracks when chemical bonds are broken. Based on this model, a theoretical analysis has determined the coupling relationship between EM energy density and charge density that could guide the development of warning strategies for mine safety.

Analyzing stress modifications in tunnels and quantifying stress: This was done by taking cross-sectional measurements along the interior of a tunnel (length: 240 m) located in the Darjeeling-Sikkim Himalayas to determine the azimuth and amplitude of maximum FEMR emission on a plane perpendicular to the tunnel length. Subsequent calculations leading to the computation of the horizontal stresses were based on the fact that the shear stresses acting on the tunnel wall are directly proportional to the maximum FEMR amplitude^26^. Lichtenberger^21,22^ first demonstrated using the FEMR technique inside a tunnel to calculate stress magnitude and orientation. His pioneering work in the Feuerberg tunnel, Southwest of Germany (Lichtenberger^21^), and in the Wald-Michelbach tunnel near the Odenwald Mountains, Germany (Lichtenberger^22^) helped to determine the azimuth as well as the magnitude of the horizontal principal stresses inside tunnels. The former study additionally revealed the presence of a fault, which led to an erratic increase in the impulse of the measured FEMR signals. In contrast, the latter study indicated the superimposition of a secondary tensional stress field in the region of measurement^21,22^. Following the calculations, although a reverse stress regime is prominent and established over the Himalayas, the results show a normal stress regime prevailing over this shallow, near-surface region of the tunnel, and the direction of maximum shear stress changes along the tunnel length. The results indicate the reactivation of a thrust plane with variable dips in the normal stress regime is causative for the modification of local stress along the tunnel. The interpretation was further explained with a simple analog model where normal reactivation of a thrust plane with changing dips was simulated.

### Future studies

For future analysis, it can be proposed that to consociate the two models as discussed in sections “FEMR nucleation stages: Fish-shape signals model” and “The time-series model”, the parameters that prove to be essential for further study as well as bridging the gap would be energy, which is calculated from Eq. (5). Furthermore, this would also connect to the crack dimensions (primarily crack area) as they follow a similar trend in their respective plots (Table 1). The amplitude data from the respective channels is obtained for both models. Hence, a Benioff strain plot can be constructed, which can provide insight into the various stages of nucleation from both models as well as provide an indirect warning of an approaching failure.

Furthermore, both models can help comprehend the FEMR activity or intensity acquired from the same earthquake generation process around the fault zone. This could establish a possible correlation between the obtained FEMR signals and the earthquake event.

As previously mentioned, the locations of the monitoring points were chosen so that they are in the vicinity of active and potentially active faults (Fig. 2). An increase in the number of monitoring points would undoubtedly enable the reproducibility of the results. Nonetheless, the current locations of the survey (1–9) provide adequate and well-rounded insight into the variation of the FEMR parameters in the Dead Sea Basin. Figures 7, 8, 9 and 10 show the plots for the different FEMR parameters, such as Benioff Strain, hits, frequency, rise time, crack dimensions, etc., and sufficiently enable the visualization of their corresponding trends before the EQ. In the future, we aim to conduct a more detailed survey in an increased number of monitoring locations around the previously studied points to assess the reproducibility of the previous data and verify the trends in the FEMR parameters. Additionally, we would analyze the diurnal variation of the FEMR parameters along the Dead Sea basin for future studies.

The multi-scale features of FEMR pulses have not yet been discerned in terms of their scale factor. In other words, a clear correlation between lab-scale and large-scale phenomena has not yet been established in this realm of FEMR studies. Hence, we are working on direct shear experiments using pre-cut samples with or without asperities by integrating the approaches above for FEMR examination. The friction phenomenon is a critical element of the pre-slip model, while the FEMR studies during friction/strike-slip are rare. Various friction experiments showed that the sizes of broken asperities agreed with the FEMR estimates received during compression tests^32,37^. The results of coal studies showed that the FEMR signals are weak, and their energy is low at the stable sliding stage, while they are generated intermittently at the stick–slip stage^81^. Hence, the ongoing experiments can further provide a fresh perspective and add value to the general parameters and the individual pulse parameters, along with their connection with the crack dimensions, which correlate adequately from the theoretical point of view and as observed from the field data. This could be a significant step in bridging the gap between lab-scale and large-scale phenomena.

The prerogative of this survey was to compare the trends of the FEMR parameters in the tectonically active region of DST before the occurrence of an Earthquake. The goal for our future studies will be to compare our data in the strike-slip fault regime with results from the convergent fault boundaries (e.g., the Himalayas), which will be the basis of our simulations.

## Conclusions

Based on the FEMR study along the Dead Sea Transform (DST), the following conclusions can be made:

- FEMR data was acquired from 9 locations along a segment of the tectonically active DST, with the last measurement taken approximately two hours before the Syrian-Turkey earthquake (M_w_ = 6.3).
- Several parameters, such as cumulative amplitudes, frequencies, rise time, hits/activity, and energies of FEMR pulses, were computed from the filtered data. These processed parameters were, in turn, utilized to infer the corresponding crack dimensions.
- Plots of various parameters, including FEMR hits, frequency, rise time, and crack dimensions, were generated to illustrate trends leading up to an earthquake. The Benioff strain plot revealed distinct stages in crack nucleation. In the first stage, FEMR hits and frequency increased while rise time and crack dimensions decreased. Energy parameters showed minimal change. The second stage saw declining FEMR hits and crack width but increasing trends in other parameters. The third stage involved rising FEMR hits, energies, and crack width, with anomalies in rise time, crack length, and cumulative area trends. Frequency decreased in this stage.
- This study provides crucial insights into analyzing FEMR parameters and their patterns before earthquakes. The hits plot exhibits a cyclic trend, with each stage of the Benioff strain plot marked by alternating high activity and silence, attributed to stress drop during crack propagation. The energy plot reveals a notable second peak in the second stage of the Benioff strain plot, corresponding to a high-stress drop. The transition between the second and third stages indicates a dip in energy, followed by a steady increase, potentially suggesting an impending earthquake event.
- In the prelude to earthquakes, the study reveals a reduction in the number of FEMR events alongside an increase in intensity, signifying the merging of fractures into larger units—a potential signal of an impending catastrophe. Additionally, the decrease in activation frequency aligns with bifurcation theory, indicating a slowing down before a transition. The novelty stems from its ability to scrutinize individual FEMR pulses, enabling the calculation of various pulse parameters, intensity, and energy, facilitating correlation with the dimensions of the originating fractures.
- Together, these results show the feasibility of FEMR measurements as a valid forecast of earthquake catastrophes.

## Data Availability

All data generated and analyzed during this study are included in the article.
